# Supplementary material for: Understanding the effectiveness of government interventions against the resurgence of COVID-19 in Europe
Source: Nat Commun. 2021 Oct 5;12:5820. doi: 10.1038/s41467-021-26013-4 (PMC8492703; doi:10.1038/s41467-021-26013-4)
Supplement: Supplementary file 2 — Reporting Summary [file 41467_2021_26013_MOESM2_ESM.pdf]

nature portfolio

Corresponding author(s): Mrinank Sharma, Sören Mindermann, Samir Bhatt, Jan Brauner

Last updated by author(s): Aug 17, 2021

Reporting Summary

Nature Portfolio wishes to improve the reproducibility of the work that we publish. This form provides structure for consistency and transparency in reporting. For further information on Nature Portfolio policies, see our Editorial Policies and the Editorial Policy Checklist.

Statistics

For all statistical analyses, confirm that the following items are present in the figure legend, table legend, main text, or Methods section.

n/a	Confirmed	
☐	☒	The exact sample size (*n*) for each experimental group/condition, given as a discrete number and unit of measurement
☐	☒	A statement on whether measurements were taken from distinct samples or whether the same sample was measured repeatedly
☒	☐	The statistical test(s) used AND whether they are one- or two-sided<br>*Only common tests should be described solely by name; describe more complex techniques in the Methods section.*
☐	☒	A description of all covariates tested
☒	☐	A description of any assumptions or corrections, such as tests of normality and adjustment for multiple comparisons
☐	☒	A full description of the statistical parameters including central tendency (e.g. means) or other basic estimates (e.g. regression coefficient) AND variation (e.g. standard deviation) or associated estimates of uncertainty (e.g. confidence intervals)
☒	☐	For null hypothesis testing, the test statistic (e.g. *F*, *t*, *r*) with confidence intervals, effect sizes, degrees of freedom and *P* value noted<br>*Give P values as exact values whenever suitable.*
☐	☒	For Bayesian analysis, information on the choice of priors and Markov chain Monte Carlo settings
☐	☒	For hierarchical and complex designs, identification of the appropriate level for tests and full reporting of outcomes
☒	☐	Estimates of effect sizes (e.g. Cohen's *d*, Pearson's *r*), indicating how they were calculated

Our web collection on statistics for biologists contains articles on many of the points above.

Software and code

Policy information about availability of computer code

Data collection	Google Chrome (various version numbers), Google Sheets (various version numbers), Python/Numpy/Pandas for validation (various version numbers).
Data analysis	NumPyro (version 0.6.0). All modelling code is available in the public github repo: https://github.com/MrinankSharma/COVID19NPISecondWave

For manuscripts utilizing custom algorithms or software that are central to the research but not yet described in published literature, software must be made available to editors and reviewers. We strongly encourage code deposition in a community repository (e.g. GitHub). See the Nature Portfolio guidelines for submitting code & software for further information.

Data

Policy information about availability of data

All manuscripts must include a data availability statement. This statement should provide the following information, where applicable:

- Accession codes, unique identifiers, or web links for publicly available datasets
- A description of any restrictions on data availability
- For clinical datasets or third party data, please ensure that the statement adheres to our policy

All data necessary for the replication of our results are publicly available on https://github.com/MrinankSharma/COVID19NPISecondWave/tree/main/data , or, in archived form, at (56). The NPI data was collected by the authors; case and death data was taken from local data sources—please see https://github.com/MrinankSharma/COVID19NPISecondWave/blob/main/data/raw_data_w_sources/sources.md. National case and death data for the third wave experiments taken from John Hopkins University https://github.com/CSSEGISandData/COVID-19, but accessed the OxCGRT tracker https://github.com/OxCGRT/covid-policy-tracker.

Field-specific reporting

Please select the one below that is the best fit for your research. If you are not sure, read the appropriate sections before making your selection.

- [x] Life sciences
- [ ] Behavioural & social sciences
- [ ] Ecological, evolutionary & environmental sciences

For a reference copy of the document with all sections, see nature.com/documents/nr-reporting-summary-flat.pdf

Life sciences study design

All studies must disclose on these points even when the disclosure is negative.

Sample size	Sample size was chosen according to a random study design. For Austria, the Czech Republic, Italy, and the Netherlands, it was feasible to collect data from the whole country (9, 14, 21, and 25 regions of analysis). For all other countries, we took a stratified random sample of 15 regions of analysis. Temporally we used all available data between 1st August 2020 and 9th January 2021. We note that, in our framework, calculations of sample size to reach a certain statistical power, as in frequentist analyses, are not necessary as our analysis is fully Bayesian.
Data exclusions	We considered several additional NPIs for inclusion, e.g. a shielding of vulnerable populations, but were unable to obtain data of sufficient quality, and thus did not include them.
Replication	We conducted many sensitivity analyses, they are reported in Figure 3 and the Supplement.
Randomization	For Austria, the Czech Republic, Italy, and the Netherlands, it was feaslble to collect data from the whole country [9, 14, 21, and 25 regions of analysis]. From each other country, we took a stratified random sample of 15 regions of analysis. The sample was stratified by the regions' number of COVID-deaths in the first wave, to ensure a sufficiently diverse sample and reduce the variance of our NPI effect estimator. To increase statistical precision and reduce the influence of imported infections. we did not include regions with fewer than 2000 reported cases during the analysis period.
Blinding	No group allocation and thus no blinding.

Reporting for specific materials, systems and methods

We require information from authors about some types of materials, experimental systems and methods used in many studies. Here, indicate whether each material, system or method listed is relevant to your study. If you are not sure if a list item applies to your research, read the appropriate section before selecting a response.

Materials & experimental systems

n/a	Involved in the study	
☒	☐	Antibodies
☒	☐	Eukaryotic cell lines
☒	☐	Palaeontology and archaeology
☒	☐	Animals and other organisms
☒	☐	Human research participants
☒	☐	Clinical data
☒	☐	Dual use research of concern

Methods

n/a	Involved in the study	
☒	☐	ChIP-seq
☒	☐	Flow cytometry
☒	☐	MRI-based neuroimaging
